# Distinct roles of Dlk1 isoforms in bi-potential differentiation of hepatic stem cells

**DOI:** 10.1186/s13287-019-1131-2

**Published:** 2019-01-15

**Authors:** Jiefang Huang, Xiaonan Zhao, Jian Wang, Yiji Cheng, Qiong Wu, Bei Wang, Fang Zhao, Lijun Meng, Yanyun Zhang, Min Jin, Huanbai Xu

**Affiliations:** 1 Institute of Pediatric Research, Children’s Hospital of Soochow University, Institutes for Translational Medicine, Soochow University, Suzhou, 215025 China; 2 Key Laboratory of Tissue Microenvironment and Tumor, Shanghai Institutes for Biological Sciences, Chinese Academy of Sciences, Shanghai, 200031 China; 3 Department of Endocrinology and Metabolism, Shanghai General Hospital, School of Medicine, Shanghai Jiao Tong University, Shanghai, 200080 China

**Keywords:** Hepatic stem cells, Dlk1, Isoforms, Differentiation

## Abstract

**Background:**

Fully understanding the developmental process of hepatic stem cells (HSCs) and the mechanisms of their committed differentiation is essential for optimizing the generation of functional hepatocytes for cell therapy in liver disease. Delta-like 1 homolog (Dlk1), primarily the membrane-bound form (Dlk1^M^), is generally used as a surface marker for fetal hepatic stem cell isolation, while its soluble form (Dlk1^S^) and the functional roles of different Dlk1 isoforms in HSC differentiation remain to be investigated.

**Methods:**

Hepatic spheroid-derived cells (HSDCs) were isolated from E12.5 mouse livers to obtain Dlk1^+^ and Dlk1^−^subpopulations. Colony formation, BrdU staining, and CCK8 assays were used to evaluate the cell proliferation capacity, and hepatic/cholangiocytic differentiation and osteogenesis/adipogenesis were used to assess the multipotency of the two subpopulations. Transformation of Dlk1^+^ cells into Dlk1^−^ cells was detected by FACS, and the expression of Dlk1 isoforms were measured by western blot. The distinct roles and regulatory mechanisms of Dlk1 isoforms in HSC differentiation were investigated by overexpressing Dlk1^M^.

**Results:**

HSDCs were capable of differentiating into liver and mesenchymal lineages, comprising Dlk1^+^ and Dlk1^−^ subpopulations. Dlk1^+^ cells expressed both Dlk1^M^ and Dlk1^S^ and lost expression of Dlk1^M^ during passaging, thus transforming into Dlk1^−^ cells, which still contained Dlk1^S^. Dlk1^−^ cells maintained a self-renewal ability similar to that of Dlk1^+^ cells, but their capacity to differentiate into cholangiocytes was obviously enhanced. Forced expression of Dlk1^M^ in Dlk1^−^ cells restored their ability to differentiate into hepatocytes, with an attenuated ability to differentiate into cholangiocytes, suggesting a functional role of Dlk1 in regulating HSC differentiation in addition to acting as a biomarker. Further experiments illustrated that the regulation of committed HSC differentiation by Dlk1 was mediated by the AKT and MAPK signaling pathways. In addition, bFGF was found to serve as an important inducement for the loss of Dlk1^M^ from Dlk1^+^ cells, and autophagy might be involved.

**Conclusions:**

Overall, our study uncovered the differential expression and regulatory roles of Dlk1 isoforms in the commitment of HSC differentiation and suggested that Dlk1 functions as a key regulator that instructs cell differentiation rather than only as a marker of HSCs. Thus, our findings expand the current understanding of the differential regulation of bi-potential HSC differentiation and provide a fine-tuning target for cell therapy in liver disease.

**Electronic supplementary material:**

The online version of this article (10.1186/s13287-019-1131-2) contains supplementary material, which is available to authorized users.

## Background

Liver transplantation is the ultimate therapy for patients with end-stage liver disease, but its application has been largely limited by the shortage of liver donors [1]. Cell transplantation has become an alternative therapy and a bridge for patients awaiting liver transplantation. Functional hepatocytes are the primary cell source for transplantation [2]. It has been demonstrated that embryonic stem cells (ESCs), induced pluripotent stem cells (iPSCs), and even fibroblasts can be reprogrammed and induced into hepatic stem cells (HSCs) and hepatocytes, largely based upon the signals that arise during liver development [3]. Therefore, further elucidation of the process and mechanisms of liver development, especially committed HSC differentiation, is essential for optimization of strategies to obtain high-quality hepatocytes with enhanced maturity and stability.

During embryonic liver development, fetal hepatic stem cells, also known as hepatoblasts, are common progenitors of hepatocytes and cholangiocytes [4]. In theory, the study of hepatoblasts facilitates the application of cell therapy for liver regeneration. Due to the overwhelming complexity in vivo, studies of hepatoblasts are usually performed ex vivo or in vitro. Identification of hepatoblast populations at different developmental stages will greatly facilitate the study of hepatic biology and reveal important signaling molecules and mechanisms crucial to hepatoblast function. At present, identification and isolation of hepatoblasts primarily depends on the expression of multiple cell surface molecules. For example, Suzuki et al. demonstrated that hepatoblasts are enriched in CD45^−^TER119^−^c-kit^−^CD29^+^CD49f^+/low^ cell or CD45^−^TER119^−^c-kit^−^CD49f^+/low^cMet^+^ cell fractions from embryonic day (E) 13.5 mouse livers [5, 6]. Nierhoff et al. identified additional markers, CD24a and Nope, that can be used to isolate hepatoblasts from E13.5 mouse livers [7]. In E12.5 livers, hepatoblasts were shown to specifically express E-cadherin, Delta-like 1 homolog (Dlk1), and Liv2 [8]. The diverse markers used in different studies suggest that hepatoblasts likely change their characteristics during the course of liver development. Nevertheless, whether these molecules serve as regulatory signals during the process or just as cellular markers needs to be explored.

Among the recognized markers of hepatoblasts, Dlk1 is highly expressed in the E10.5 liver bud, and expression continues until E16.5 [9]. Moreover, Tanimizu et al. successfully isolated hepatoblasts from E14.5 mouse livers based on the expression of Dlk1 instead of that of grouped markers, indicating that acquisition of Dlk1^+^ cells might be a more convenient way to obtain hepatoblasts [9–11]. Interestingly, alternatively spliced transcripts of Dlk1 have been described that encode either a membrane-tethered Dlk1 (Dlk1^M^) or full-length Dlk1 (Dlk1^SM^) isoform, which contains a juxtamembrane motif for cleavage by extracellular proteases to further generate the soluble isoform of Dlk1 (Dlk1^S^) [12]. Generally, the Dlk1 isoform used for hepatoblast isolation is Dlk1^M^, and the existence of Dlk1^S^ in hepatoblasts has been neglected. Although the roles of Dlk1 in liver development remain unknown, its regulatory function in several differentiation processes has been clarified, including adipogenesis [13], osteogenesis [14], and neurogenesis [15]. Furthermore, Dlk1^S^ also plays a role in differentiation, such as myogenesis [16]. Taken together, these studies suggest that Dlk1 may function as more than a biomarker of hepatoblasts and might act as a regulator of HSC differentiation.

In the present study, we found that hepatic spheroid-derived cells (HSDCs) from E12.5 mouse fetal livers exhibited multipotency and contained two subpopulations, Dlk1^+^ cells and Dlk1^−^ cells. Dlk1^+^ cells expressed both Dlk1^M^ and Dlk1^S^, while Dlk1^−^ cells contained only the soluble isoform Dlk1^S^. More importantly, Dlk1^+^ cells could lose expression of Dlk1^M^ to become Dlk1^−^ cells. Functionally, Dlk1^−^ cells maintained self-renewal capability and acquired enhanced potency to differentiate into cholangiocytes compared with Dlk1^+^ cells, making them cholangiocyte precursor-like cells. Overexpression of Dlk1^M^ in Dlk1^−^ cells upregulated hepatic differentiation and inhibited cholangiocytic differentiation through regulation of AKT and MAPK signaling pathways. Additionally, bFGF was responsible for the transformation of Dlk1^+^cells to Dlk1^−^ cells, and autophagy might be involved. Thus, our study indicated that Dlk1 isoforms were differentially expressed and played distinct roles during committed HSC differentiation, providing clues and evidence for optimizing cell therapy strategies for liver disease.

## Methods

### Animals

C57BL/6 mice (7–8 weeks old) were purchased from the Shanghai Laboratory Animal Center of the Chinese Academy of Sciences and housed under specific-pathogen-free conditions in the animal center of Shanghai Jiao Tong University School of Medicine (Shanghai, China). All animal procedures were approved by the Animal Welfare and Ethics Committee of Shanghai Jiao Tong University School of Medicine.

### Enrichment of HSDCs from fetal mouse liver

HSDCs were enriched as previously described [17] with some modifications. Briefly, fetal liver tissues were removed from pregnant C57BL/6 mice at embryonic day 12.5. Dissociated liver cells were centrifuged at 500 rpm for 3 min in cold phosphate-buffered saline (PBS) and then cultured on six-well ultralow attachment plates (Corning, Corning, NY, USA) at a density of 5 × 10^5^ cells per milliliter in standard Dulbecco’s modified Eagle’s medium/F12 (Sigma, St Louis, MO, USA) supplemented with B27 (Gibco, Grand Island, NY, USA), insulin-transferrin-selenium X (ITS-X, Gibco), 10 mmol/L HEPES (Gibco), antibiotics, 20 ng/mL epidermal growth factor (EGF; Sigma), 20 ng/mL basic fibroblast growth factor (bFGF; R&D Systems, Minneapolis, MN, USA), and 20 ng/mL hepatocyte growth factor (HGF; Sigma). Hepatic spheroids were collected at day 6 by centrifugation at 300 rpm for 2 min and plated on type I collagen-coated dishes (Becton Dickinson, San Jose, CA, USA). After growing to subconfluency, the HSDCs were passaged using Accutase (Gibco), and the HGF concentration was reduced to 10 ng/mL after plating on collagen-coated dishes.

### Mesenchymal lineage differentiation

The differential capacity of HSDCs into mesenchymal lineages, adipocytes and osteocytes, was determined by applying adipogenic (MUBMX-90031, Cyagen, China) and osteogenic (MUBMX-90021, Cyagen) induction medium according to the manufacturer’s instructions. Adipogenesis was assessed by Oil Red O staining, and osteogenesis was assessed by Alizarin Red S staining.

### Hepatic differentiation

Hepatic differentiation was performed according to the 2D or 3D induction method as previously described [18]. In the 2D protocol, cells plated in type I collagen-coated plates were cultured in hepatic induction medium (DMEM supplemented with 20 ng/mL oncostatin M, 50 mg/mL ITS-X, 10 μg/mL insulin, and 1 μmol/L dexamethasone). The medium was refreshed twice a week. After 3 weeks of induction, hepatogenesis was assessed by PAS staining and the expression of liver-associated genes (ALB, G6P, TAT, TO) determined by real-time PCR. In the 3D protocol, cells were digested from collagen-coated plates and resuspended in hepatic induction medium and then transferred to a 24-well plate coated with Matrigel. The medium was refreshed every 2 days, and cells were collected at the indicated times for further experiments.

### Cholangiocytic differentiation

Cholangiocytic differentiation was performed as previously described [19]. Briefly, cells (1 × 10^5^ cells per 35-mm dish) resuspended in 1 mL DMEM/F12 were mixed with 1 mL of collagen gel solution (Collagen Type I-A Gel Culture Kit; Nitta Gelatin, Osaka, Japan). These cell mixtures were plated onto a basal layer of collagen. After 30 min of incubation at 37 °C, the cells were cultured in 2 mL DMEM supplemented with 10% FBS, 1× ITS-X, 20 ng/mL HGF, and 50 ng/mL tumor necrosis factor-α (TNF-α). Cells were induced for 8–10 days, and the medium was refreshed every 3 days.

### Flow cytometry

Cells were washed with PBS and resuspended in PBS with 2.5% FBS. Cells were simultaneously stained with FITC-conjugated CD45, c-kit, and TER119 mAbs, APC-conjugated CD49f mAb, and PercpCy5.5-conjugated CD29 mAb at 4 °C for 30 min. In addition, they were incubated with PE-conjugated Dlk1 (all from eBioscience, San Diego, CA, USA). The labeled cells were analyzed using a FACSVantage cell sorter (BD, San Jose, CA, USA). Gating was implemented based on negative-control staining profiles. In some experiments, the PE-conjugated Dlk1-labeled cells were sorted into positive and negative subpopulations for further studies.

### Immunofluorescence

Cells were embedded in optimum cutting temperature (OCT, Sakura Finetek, Japan) compound and cut into 5-μm-thick sections for staining as previously described [20]. Briefly, after being washed with PBS, cells were fixed in 4% PFA at room temperature for 30 min and permeabilized with 0.5% Triton-X100 for 10 min. Cells were blocked with 1% BSA for 1 h at 37 °C, and antibodies specific for α-fetoprotein (AFP), albumin (ALB), cytokeratin 19 (CK19), or LC3II (all from Abcam, Cambridge, MA, USA) were added and incubated with cells at 4 °C overnight. Then, Alexa 488-conjugated or Alexa 555-conjugated secondary antibodies were applied for 1 h at 37 °C in the dark, followed by DAPI staining.

### Lentiviral vector construction and cell transduction

Overexpression of Dlk1^M^ in Dlk1^−^ cells was achieved with lentivirus based on a pLVX-IRES-zsGreen (Clontech, Japan) vector containing the Dlk1^M^ sequence amplified from mouse cDNA with the forward primer 5′-CCGGAATTCATGATCGCGACCGGAGCCCT-3′ and reverse primer 5′-CGCGGATCCTTAGATCTCCTCATCACCA-3′. 293T cells were transfected with mock or Dlk1^M^ vector using Lipofectamine 2000 (Invitrogen, Carlsbad, CA, USA). Lentivirus was collected 3 days later and used to transduce Dlk1^−^ cells using Lipofectamine 2000 as previously described [21].

### RT-PCR and quantitative real-time PCR

Total RNA was extracted and subsequently reverse-transcribed using a Reverse Transcription System (DRR036A, Takara, Shiga, Japan). RT-PCR was performed using Premix Taq™ DNA Polymerase (R004A, Takara), and the PCR products were analyzed via agarose gel electrophoresis. Quantitative real-time PCR was performed using SYBR Green PCR mix (4913914001, Roche, Basel, Switzerland) on an ABI Prism® 7900HT Sequence Detection System (Applied Biosystems, Foster City, CA, USA). GAPDH was used as the internal control to normalize for differences in the amount of total RNA in each sample. The primer sequences are listed in Additional file 1: Table S1.

### Immunoblotting

Cells were lysed with ice-cold RIPA buffer (89900, Pierce, MA, USA) containing protease and phosphatase inhibitors (Roche, 04693159001). The lysates were fractionated by SDS-PAGE and analyzed by immunoblotting with specific antibodies against Dlk1 (Abcam); phosphorylated and total AKT, ERK1/2, and p38; and LC3I/II, GAPDH, and β-actin (all from Cell Signaling Technology (CST; MA, USA)). After incubation with HRP-conjugated goat anti-rabbit IgG, the immunoreactive bands were visualized with ECL Plus western blotting detection reagents (Millipore, MA, USA).

### Clonogenic assay

Dlk1^+^ and Dlk1^−^ cells were plated at 300 cells per well in six-well collagen I-coated plates. After culture for 10–12 days, the cells were washed with PBS and fixed with 4% PFA, and the colonies were stained with crystal violet. The number of visible colonies was determined.

### Proliferation assay

Dlk1^+^ and Dlk1^−^ cells were seeded at a density of 1 × 10^3^ cells per well in 96-well plates. Cell growth was then analyzed every day using the Cell Counting Kit-8 (CCK8) assay (Dojindo, Kumamoto, Japan) according to the manufacturer’s instructions [18]. Dlk1^+^ and Dlk1^−^ cells were plated at 1 × 10^5^ cells per well in six-well collagen I-coated plates. After 48 h, BrdU was added to cells at a final concentration of 10 μmol/L for 4 h. BrdU labeling was then determined via FACS according to the manufacturer’s protocol.

### Statistical analysis

All measurement data are presented as the mean ± SEM. SPSS software, version 20 (IBM, Armonk, NY), was used for all statistical analyses. Significant differences were evaluated using Mann-Whitney test. One-way ANOVA with post Dunn’s multiple comparisons test or two-way ANOVA with post Bonferroni’s multiple comparisons test was used to determine multigroup differences. Significance is expressed as *P < 0.05, **P < 0.01, or ***P < 0.001.

## Results

### HSDCs from E12.5 fetal mouse livers comprised Dlk1^+^ and Dlk1^−^ subpopulations

**Fig. 1:**
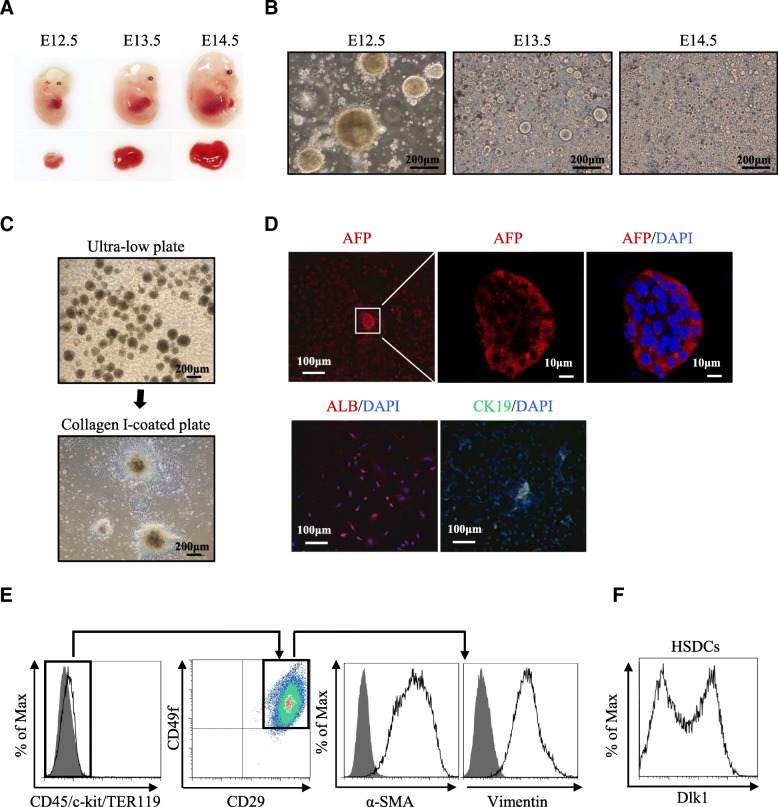
HSDCs from E12.5 fetal mouse livers contained Dlk1^+^ and Dlk1^−^ subpopulations. **a** Representative pictures of fetal livers dissected form E12.5, E13.5, and E14.5 mice (*n* = 6). **b** Cells were isolated from fetal livers, and hepatic spheroids were obtained from floating cultures on ultralow attachment plates for 6 days. **c** E12.5 spheroids were collected from ultralow attachment plates and plated onto collagen I-coated plates. **d** E12.5 HSCDs were collected, and the expression of AFP, ALB, and CK19 was detected via immunofluorescence. **e** The immunophenotype of E12.5 HSDCs was assessed with FACS. **f** The expression of Dlk1 in E12.5 HSDCs was detected by FACS. Representative images from one of three experiments are shown

Fetal livers at different embryonic days were dissected, and cells were cultured in a serum-free culture system to facilitate enrichment of HSCs, a technique which has been used previously to amplify stem/progenitor cells from fetal livers [17]. As shown in Fig. 1a, the size of mouse fetal livers changed rapidly during embryonic development, suggesting different stages of HSCs with distinct characteristics at different embryonic days. Spheroids from E12.5 fetal livers grew faster and larger than those from E13.5 and E14.5 fetal livers, increasing to a diameter of approximately 100–300 μm within 4 days (Fig. 1b). When the spheroids were plated on type I collagen-coated dishes, they formed monolayer colonies (Fig. 1c). Immunofluorescence analysis of these cells showed positive expression of AFP, ALB, and CK19 (Fig. 1d), which indicated that the sphere-forming cells from E12.5 fetal livers are heterogeneous HSCs as reported [17]. After cells grew to subconfluency, accutase was used to digest cells into a single-cell suspension to ensure viability and long-term culture. Then, flow cytometry was carried out to characterize the immunophenotype of HSDCs. As shown in Fig. 1e, these cells exhibited a CD45^−^c-kit^−^TER119^−^CD29^+^CD49f^+^ phenotype, the same reported immunophenotype of fetal hepatic stem cells [5]. Unexpectedly, all the CD45^−^c-kit^−^TER119^−^CD29^+^CD49f^+^ cells were also positive for the mesenchymal markers Vimentin and α-SMA (Fig. 1e). Furthermore, Dlk1 was differentially expressed in these cells, as shown in Fig. 1f, demonstrating that HSDCs from E12.5 fetal mouse livers consisted of Dlk1^+^ and Dlk1^−^ subpopulations.

### HSDCs from E12.5 livers possessed multidifferentiation potency

**Fig. 2:**
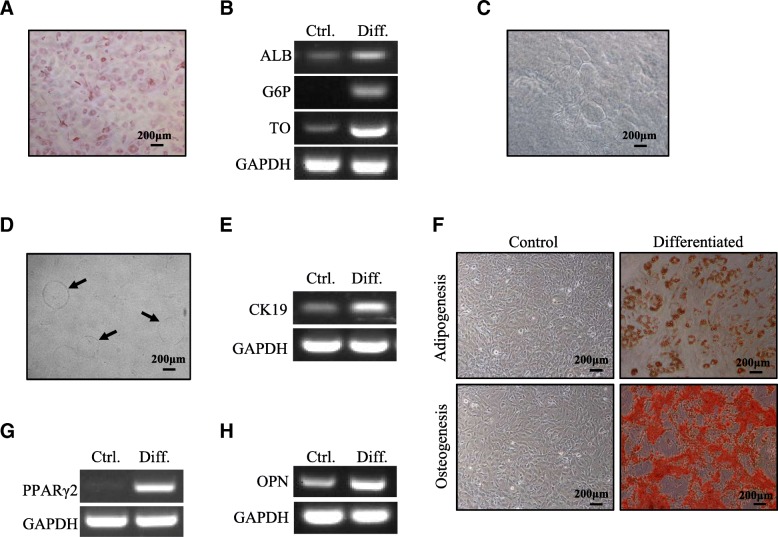
HSDCs from E12.5 fetal mouse livers possessed multidifferentiation potency. **a**, **b** HSDCs were cultured in hepatic differentiation medium for 3 weeks, and then, PAS staining was performed to detect synthesized glycogen (**a**), and the expression of hepatic markers (ALB, G6P, TO) was measured via RT-PCR (**b**). **c**–**e** HSDCs were induced into cholangiocytes in three-dimensional culture for 8 days, and the formation of cyst (**c**) and bile duct-like structures is indicated by arrows (**d**); the expression of the cholangiocytic marker CK19 was measured via RT-PCR (E). **f**–**h** HSDCs were cultured in either adipogenic or osteogenic medium. **f** Oil Red O was used to evaluate adipogenesis, and Alizarin Red S was used to detect osteogenesis. **g** Expression level of adipocyte marker PPAR-γ2 was assessed by RT-PCR. **h** The expression level of the osteocyte marker OPN was assessed using RT-PCR. Crtl. control, Diff. differentiated. Representative images from one of three experiments are shown

Considering the expression of hepatic and mesenchymal markers by HSDCs, we investigated whether these cells had the capacity to develop into liver and mesenchymal lineages. After induction with hepatic differentiation medium containing oncostatin M (OSM) for 3 weeks, these cells displayed glycogen-containing vesicles within the cytoplasm indicated by PAS staining (Fig. 2a). RT-PCR analysis revealed obvious expression of the mature hepatocyte-specific genes ALB, glucose-6-phosphatase (G6P), and tryptophan-2,3-oxygenase (TO) (Fig. 2b). Under cholangiocyte differentiation conditions, HSDCs formed cyst and bile duct-like structures in three-dimensional culture with collagen (Fig. 2c, d). CK19, a marker of cholangiocytes, was also significantly increased (Fig. 2e). Moreover, HSDCs were capable of differentiating into mesenchymal lineages, adipocytes (Fig. 2f, g) and osteocytes (Fig. 2f, h), based on morphology staining and gene expression. Altogether, HSCDs with Dlk1^+^ and Dlk1^−^ subpopulations were found to possess multidifferentiation potency.

### Dlk1^+^ HSDCs could lose Dlk1^M^ and transform into Dlk1^−^ cells

**Fig. 3:**
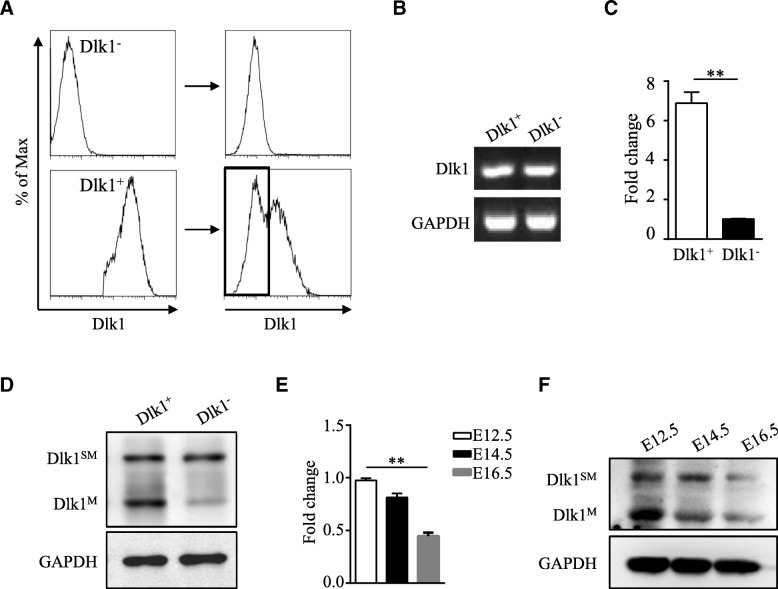
Dlk1^+^ cells in HSDCs could lose Dlk1^M^ and transform into Dlk1^−^ cells. **a**–**d** Dlk1^+^ and Dlk1^−^ cells were sorted from E12.5 HSDCs via FACS and then cultured on type I collagen-coated dishes. After three passages, the cells were collected, and the expression of Dlk1 was detected by FACS (**a**). Dlk1^+^ cells and Dlk1^−^ cells derived from Dlk1^+^ cells were sorted and collected via FACS, and then, RT-PCR (**b**) and quantitative real-time PCR (**c**) were performed to measure the mRNA expression level of Dlk1, and western blotting (**d**) was used to detect the protein expression level of Dlk1 isoforms. HSDCs isolated from E12.5, E14.5, and E16.5 mouse fetal livers were collected, and the expression level of Dlk1 was determined by quantitative real-time PCR (**e**) and western blotting (**f**). Representative images from one of three experiments are shown, and the data are shown as the mean ± SEM of three independent experiments. **P < 0.01 (Mann-Whitney test in **c** and one-way ANOVA with post Dunn’s multiple comparisons test in **e**)

To further characterize and explore differences between Dlk1^+^ and Dlk1^−^ cells, cells were sorted via FACS. Interestingly, it was found that Dlk1^+^ cells could give rise to Dlk1^−^ cells during passaging, while Dlk1^−^ cells only generated Dlk1^−^ cells, even after multiple passages (Fig. 3a). Then, we tried to confirm the expression of Dlk1 in these two cell populations and were surprised to find that Dlk1^−^ cells still expressed Dlk1 at the mRNA level (Fig. 3b), although they displayed a lower expression level than Dlk1^+^ cells (Fig. 3c). Further western blotting analysis revealed that Dlk1^+^ cells expressed both the isoform Dlk1^SM^ which can produce Dlk1^S^ and the membrane-bound isoform Dlk1^M^ while Dlk1^−^ cells only expressed Dlk1^SM^ (Fig. 3d), suggesting that Dlk1^−^ cells were not completely Dlk1 negative but expressed an alternative soluble isoform of Dlk1. Since Dlk1 is reported to be highly expressed in fetal livers from E10.5 and expression begins to decrease on E16.5 [9], we evaluated whether the expression of Dlk1 isoforms changes during liver development. As shown in Fig. 3e and f, the mRNA expression of Dlk1 was significantly downregulated on E16.5, and the Dlk1^M^ level decreased along with the embryonic day, similar to the loss of Dlk1^M^ in Dlk1^+^ cells during in vitro passaging. Overall, these data demonstrate that Dlk1^+^ cells in HSDCs can convert to Dlk1^−^ cells through loss of Dlk1^M^ expression but still contain Dlk1^S^.

### Loss of Dlk1^M^ in HSDCs gave rise to cholangiocyte progenitor-like cells

**Fig. 4:**
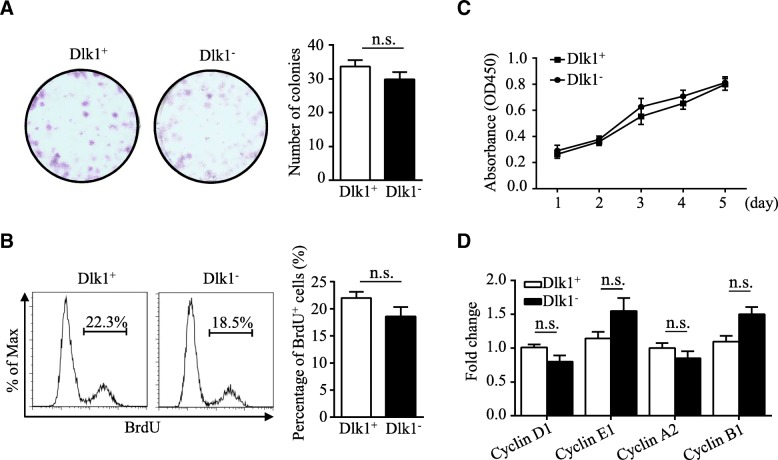
Dlk1^−^ cells and Dlk1^+^ cells had similar proliferative capacity. Dlk1^+^ cells and Dlk1^−^ cells derived from Dlk1^+^ cells were sorted using FACS. **a** Dlk1^+^ or Dlk1^−^ cells were plated in six-well collagen-coated plates at a density of 300 cells per well. After 10–12 days of culture, the colonies were stained with crystal violet, and the number of colonies was determined. **b** Dlk1^+^ and Dlk1^−^ cells were plated at 1 × 10^5^ cells per well in six-well collagen-coated plates. After 48 h, BrdU was added to cells at a final concentration of 10 μmol/L for 4 h, and then, BrdU labeling was assessed via FACS, and the proportion of BrdU-positive (BrdU^+^) cells was calculated. **c** Dlk1^+^ and Dlk1^−^ cells were seeded at a density of 1 × 10^3^ cells per well in 96-well plates. Cell growth was measured daily using CCK8 assays. **d** The expression levels of cyclin D1, cyclin E1, cyclin A2, and cyclin B1 in Dlk1^+^ cells and Dlk1^−^ cells were measured via quantitative real-time PCR. Representative images from one of three experiments are shown, and the data are shown as the mean ± SEM of three independent experiments (Mann-Whitney test in **a** and **b**, one-way ANOVA with post Dunn’s multiple comparisons test in **c**, two-way ANOVA with post Bonferroni’s multiple comparisons test in **d**). n.s. not significant

Next, the role of Dlk1 isoforms in HSC biological behavior was determined. As shown in Fig. 4a, Dlk1^−^ cells had a colony-formation capability similar to that of Dlk1^+^ cells. BrdU detection and CCK8 assays further confirmed that there was no significant difference in the proliferation efficiency between Dlk1^+^ cells and Dlk1^−^ cells (Fig. 4b, c). Furthermore, the expression levels of the cell cyclin-dependent kinases cyclin D1, cyclin E1, cyclin A2, and cyclin B1, which play crucial roles in different cell cycle stages remained unchanged between Dlk1^+^ cells and Dlk1^−^ cells (Fig. 4d). Hence, it was demonstrated that Dlk1^+^-derived Dlk1^−^ cells maintain their proliferation capability.

**Fig. 5:**
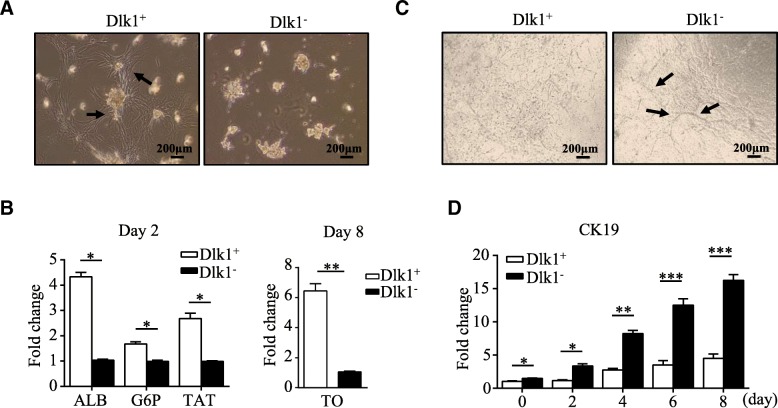
Dlk1^−^ cells appeared to be cholangiocyte progenitor-like cells. Dlk1^+^ cells and Dlk1^−^ cells derived from Dlk1^+^ cells were sorted using FACS. **a**, **b** Dlk1^+^ cells and Dlk1^−^ cells were induced for hepatic differentiation with Matrigel and OSM. Phase-contrast images presented the morphologies of the cultured Dlk1^+^ cells and Dlk1^−^ cells on Matrigel after 8 days (**a**). Arrows indicate differentiated cells. **b** Cells engaged in hepatic differentiation for the indicated time were collected, and the expression of the hepatic maturation markers ALB, G6P, and TAT was determined on day 2 and TO expression was measured on day 8 via quantitative real-time PCR. **c**, **d** Dlk1^+^cells and Dlk1^−^ cells were induced for cholangiocytic differentiation in collagen gel-embedded cultures. Phase-contrast images show representative views of branching structures derived from Dlk1^−^ cells compared with Dlk1^+^ cells (**c**). Arrows indicate bile duct-like branching structures. Cells engaged in cholangiocytic differentiation for the indicated times were collected, and the expression of the cholangiocyte marker CK19 was determined with quantitative real-time PCR (**d**). Representative images from one of three experiments are shown, and the data are shown as the mean ± SEM of three independent experiments. *P < 0.05; **P < 0.01; ***P < 0.001 (Mann-Whitney test and two-way ANOVA with post Bonferroni’s multiple comparisons test)

Dlk1^+^ cells have been confirmed as hepatoblasts with the bi-potential to differentiate into hepatocytes and cholangiocytes, and thus, we next investigated whether the differentiation potency would change when the cells transformed into Dlk1^−^ cells. After culture in hepatic differentiation medium for 8 days, there were plenty of cells spreading out from Dlk1^+^ cell colonies, but none formed Dlk1^−^ cell colonies (Fig. 5a). Quantitative real-time PCR analysis revealed higher expression levels of the mature hepatocyte-specific genes ALB, G6P, and tyrosine amino transferase (TAT) on induction day 2 and increased expression of the later mature hepatocyte marker TO on day 8 in Dlk1^+^ cells (Fig. 5b). In contrast, when cultured in cholangiocytic differentiation medium, Dlk1^−^ cells differentiated into cholangiocyte-like cells more efficiently, featuring larger representative branching structures, than Dlk1^+^ cells (Fig. 5c). Quantitative real-time PCR also revealed that Dlk1^−^ cells displayed higher expression of CK19 than Dlk1^+^ cells during the differentiation process (Fig. 5d). Thus, these results demonstrate that Dlk1^−^ cells derived from the loss of Dlk1^M^ in Dlk1^+^ cells tended to differentiate into cholangiocytes rather than hepatocytes, indicating properties similar to those of cholangiocyte progenitors.

### Dlk1^M^ served as a regulator of committed HSC differentiation by affecting AKT and MAPK signaling

**Fig. 6:**
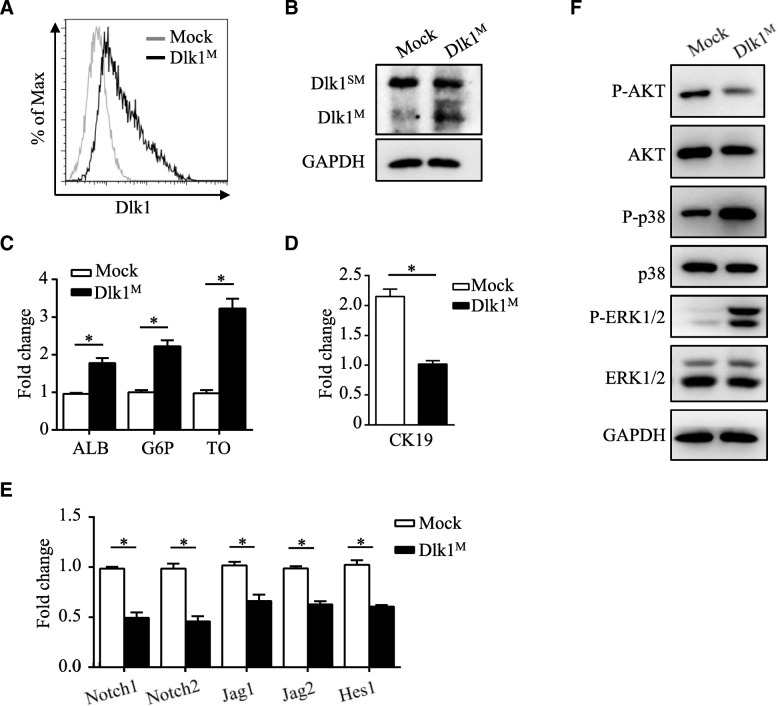
Dlk1^M^ served as a regulator of differentiation by affecting AKT and MAPK signaling. Dlk1^−^ cells were infected with mock- or Dlk1^M^-expressing viruses, and the expression of Dlk1^M^ was detected by FACS (**a**) and western blotting (**b**). **c** Dlk1^−^ cells infected with mock- or Dlk1^M^-expressing viruses were cultured under hepatic induction conditions with OSM and Matrigel. The expression levels of ALB, G6P, and TO were detected using quantitative real-time PCR. **d** Dlk1^−^ cells infected with mock- or Dlk1^M^-expressing viruses were cultured under cholangiocytic induction conditions. The expression level of CK19 was detected using quantitative real-time PCR. **e**, **f** Dlk1^−^ cells infected with mock- or Dlk1^M^-expressing viruses were cultured for 4 days under normal culture conditions. The expression levels of Notch receptors and ligands were determined by quantitative real-time PCR (**e**), and signaling pathways were analyzed via western blotting (**f**). Representative images from one of three experiments are shown, and the data are shown as the mean ± SEM of three independent experiments. *P < 0.05 (Mann-Whitney test in **d** and two-way ANOVA with post Bonferroni’s multiple comparisons test in **c** and **e**)

To determine whether Dlk1 isoforms distinctly regulate differentiation of hepatoblasts, we overexpressed Dlk1^M^in Dlk1^+^ cell-derived Dlk1^−^ cells (Fig. 6a, b). Then, the effect of Dlk1^M^ overexpression on Dlk1^−^ cell differentiation was analyzed. When cultured with hepatic differentiation medium, cells with Dlk1^M^ overexpression showed markedly increased expression of the hepatocyte markers ALB, G6P, and TO (Fig. 6c). As expected, overexpression of Dlk1^M^ reduced the expression of CK19 during cholangiocytic differentiation (Fig. 6d). Moreover, Notch signaling, which plays a critical role in cholangiocytic differentiation, was also downregulated in Dlk1^M^-overexpressing cells (Fig. 6e). Therefore, it was suggested that Dlk1^M^ overexpression could reverse the differentiation specificity of Dlk1^−^ cells to some extent. To further explore the signaling pathways mediating the effect of Dlk1^M^ on HSC differentiation, we found that AKT activation was downregulated in the Dlk1^M^-overexpressing Dlk1^−^ cells while MAPKs p38 and ERK1/2 were activated (Fig. 6f). Therefore, these results suggest that Dlk1^M^ affects the differentiation characteristics in Dlk1^+^-to-Dlk1^−^ transformation through the AKT and MAPK signaling pathways.

### bFGF induced the loss of Dlk1^M^

**Fig. 7:**
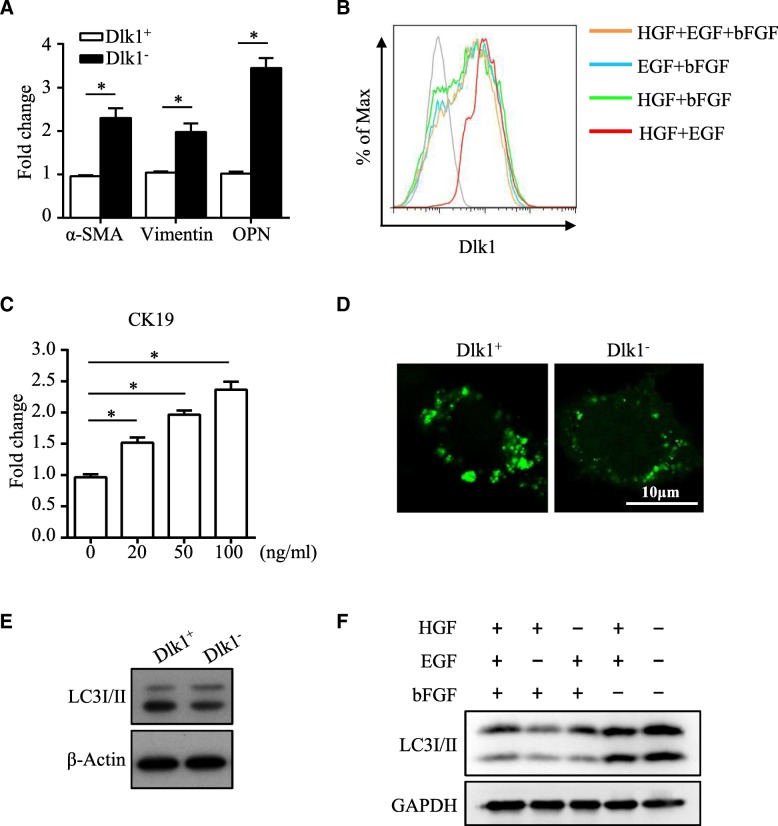
bFGF played a part in inducing the loss of Dlk1^M^. **a** The expression levels of α-SMA, Vimentin, and OPN in Dlk1^+^ and Dlk1^−^ cells were measured via quantitative real-time PCR. **b** Dlk1^+^ cells were plated in six-well collagen I-coated plates at 1 × 10^5^ cells per well and cultured with different sets of growth factors. After 6 days, the expression of Dlk1 was measured via FACS. **c** Dlk1^+^ cells were induced to cholangiocytic differentiation with the addition of increasing amounts of bFGF. After 6 days, the expression level of CK19 was analyzed via quantitative real-time PCR. **d** The autophagy marker LC3II (green) was detected in Dlk1^+^ and Dlk1^−^ cells using immunofluorescence. **e** The expression level of LC3I/II in Dlk1^+^ and Dlk1^−^ cells was determined by western blot. **f** Dlk1^+^ cells were plated in six-well collagen I-coated plates and cultured with different sets of growth factors for 6 days. The expression level of LC3I/II was determined by western blot. Representative images from one of three experiments are shown, and the data are shown as the mean ± SEM of three independent experiments. *P < 0.05 (two-way ANOVA with post Bonferroni’s multiple comparisons test in **a** and Mann-Whitney test in **c**)

As Dlk1^M^ was found to play a functional role in HSC differentiation, it is significant to determine the cause of the loss of Dlk1^M^. Considering that HSDCs containing Dlk1^+^ cells and Dlk1^−^ cells possess EMT characteristic, we analyzed the EMT state in Dlk1^+^ cells and Dlk1^−^ cells by detecting the expression levels of EMT-related genes. The mesenchymal markers α-SMA, Vimentin, and OPN exhibited enhanced expression in Dlk1^−^ cells compared with Dlk1^+^ cells, indicating that Dlk1^−^ cells might possess more mesenchymal features than Dlk1^+^ cells (Fig. 7a). The commitment of endoderm cells to the liver is dictated by several crucial cytokines, which also have important impacts on the process of EMT [22, 23]. Thus, we supposed that factors regulating EMT might be involved in the transformation of Dlk1^+^ to Dlk1^−^ cells. As shown in Fig. 7b, the transformation almost did not occur when bFGF was removed from the culture system. Furthermore, the cholangiocytic differentiation degree of Dlk1^+^ cells was enhanced with the addition of increasing amounts of bFGF (Fig. 7c). Altogether, these results suggest that bFGF induces the loss of Dlk1^M^ to promote the transformation of Dlk1^+^ cells into Dlk1^−^ cells. Autophagy, which negatively regulates cholangiocytic differentiation [24], has been found to be inhibited by bFGF [25], indicating its possible role in the bFGF-regulated transformation of Dlk1^+^ cells. Indeed, we found that autophagy was decreased in Dlk1^−^ cells derived from Dlk1^+^ cells (Fig. 7d, e), consistent with the enhanced cholangiocytic differentiation capability. Furthermore, autophagy was inhibited in the culture system with bFGF, while cells not exposed to bFGF maintained a higher level of autophagy (Fig. 7f). Consequently, bFGF was found to be responsible for the loss of Dlk1^M^ through autophagy regulation.

## Discussion

Encoded by a paternally imprinted gene located on human chromosome 14 and chromosome 12 in mice and highly expressed during embryonic development, Dlk1 has been confirmed to include membrane-bound and soluble isoforms [12, 13]. To date, Dlk1 serves as a mature surface marker to isolate Dlk1^+^ HSCs, but its function and the co-existence of Dlk1^M^ and Dlk1^S^ isoforms in HSCs remain to be explored [10, 11]. In the current study, we identified for the first time the expression characteristics of Dlk1 isoforms in multipotential HSDCs from mouse fetal livers. Given the different differentiation capacities of the Dlk1^+^ population expressing both Dlk1^M^ and Dlk1^S^ and the Dlk1^−^ population with the Dlk1^S^ isoform, it is suggested that the expression pattern of Dlk1^M^ and Dlk1^S^ might distinguish HSCs at different stages and lead to discrepant differential capacity, which also suggests distinct roles of Dlk1 isoforms in HSC differentiation.

The fact that Dlk1^−^ cells displayed inhibited hepatic differentiation but enhanced cholangiocytic differentiation and overexpressing Dlk1^M^ restored the hindered hepatic differentiation indicates that Dlk1^M^ might dominate HSC differentiation into hepatocytes while Dlk1^S^ plays a driving role in cholangiocytic lineage differentiation. Recently, distinct functions of Dlk1 isoforms have been demonstrated in several cell types accompanied by the co-existence of Dlk1^M^ and Dlk1^S^. It has been confirmed that only soluble Dlk1^S^ acts as an inhibitor of adipogenesis and prevents the differentiation of murine preadipocytes into mature adipocytes, while membrane Dlk1^M^ was shown to restrict adipose tissue size by inhibiting preadipocyte proliferation [26, 27]. During myogenesis, Dlk1^M^ enhances myotube formation and hypertrophy, while Dlk1^S^ inhibits myocyte differentiation and myotube formation, demonstrating that the regulation of Dlk1 isoforms is critical for normal muscle development [16]. In addition, Dlk1^S^ was found to be secreted by niche astrocytes, whereas Dlk1^M^ was present on neural stem cells and is required for the inductive effect of Dlk1^S^ on self-renewal [15]. Thus, different forms of Dlk1 have distinctive effects on self-renewal and differentiation processes in stem cells, indicating that homeostasis between Dlk1 isoforms is crucial for stem cell functions and development.

Although the molecular mechanisms by which lineage restriction of HSCs into hepatocytes and cholangiocytes is regulated have been identified, the specific signaling cascades influenced by Dlk1 isoforms in HSC differentiation remain unknown. Nevertheless, studies have shown that Dlk1 activates HSCs via Wnt pathways and epigenetic repression of Pparγ [28]. Moreover, AMPK, AKT, and MAPK pathways can be activated by Dlk1 [29–32], among which AKT and MAPK signaling have been demonstrated to take part in promoting cholangiocytic differentiation of hepatoblasts [10], thus indicating the involvement of AKT and MAPK signaling in Dlk1-regulated HSC differentiation. The results of our study show that Dlk1^M^ re-expression in Dlk1^−^ cells significantly downregulated AKT but upregulated MAPK signaling, including p38 and ERK1/2, providing further evidence. With regard to how Dlk1^M^ transforms downstream signals, Kim et al. showed that the cytoplasmic domain of Dlk1^M^ is required for its function in maintaining both clonogenicity and tumorigenicity of neuronal tumor cells [33]. However, further mechanistic studies under well-defined conditions are needed to understand the molecular mechanisms underlying Dlk1^M^ signaling transduction and the regulatory mechanisms of Dlk1^S^ in cholangiocytic differentiation.

Previous studies have demonstrated that mouse fetal liver stroma consists of cells in an EMT state [34]. Additionally, multipotent progenitor cells capable of differentiating into liver and mesenchymal lineages were also found in human fetal livers [35]. We found that HSDCs obtained from E12.5 livers spontaneously carried both epithelial and mesenchymal characteristics by expressing several marker proteins. Moreover, when Dlk1^+^ cells lost Dlk1^M^ and transformed into Dlk1^−^ cells, the expression levels of the mesenchymal markers α-SMA, Vimentin, and OPN increased, suggesting that EMT might play a role during this process. Growth factors, including bFGF, EGF, and HGF, are recognized to be essential for liver differentiation, and their blockade would impair normal liver development [36, 37]. Meanwhile, these growth factors are involved in the induction of EMT [22, 23]. Therefore, the effects of growth factors on the transformation of Dlk1^+^ to Dlk1^−^ cells were assessed, and bFGF was identified as an indispensable factor in inducing the absence of Dlk1^M^ and promoting Dlk1^+^-to-Dlk1^−^ development. It has been reported that autophagy is decreased during cholangiocytic differentiation, which contributes to cholangiocytic differentiation and morphogenesis by inhibiting the Notch1 signaling pathway [24]. Furthermore, the inhibitory effect of bFGF on autophagy has been clarified, and PI3K/Akt-mTOR signaling was found to be involved [25, 38, 39]. In the current study, autophagy was found decreased in Dlk1^−^ cells compared with Dlk1^+^ cells and downregulated by bFGF addition, consistent with the effect of bFGF on the loss of Dlk1^M^. Altogether, the transformation of Dlk1^+^ to Dlk1^−^ cells could be induced by bFGF, whose molecular mechanisms as well as the role of autophagy should be further investigated.

## Conclusions

In summary, we characterized Dlk1^+^ and Dlk1^−^ subpopulations in multipotent HSDCs from E12.5 mouse fetal livers and found that Dlk1^+^ cells could transform into Dlk1^−^ cells by losing Dlk1^M^ while preserving Dlk1^S^. Thus, we demonstrated distinct roles of Dlk1 isoforms in committed HSC differentiation, revealed by decreased hepatic differentiation but enhanced cholangiocytic differentiation in Dlk1^−^ cells, which could be reversed by Dlk1^M^ overexpression. In addition, bFGF was found to be the inducement underlying the occurrence of the Dlk1^+^-to-Dlk1^−^ transformation. Therefore, these intriguing results indicate that Dlk1 might be a regulator of HSC differentiation and liver development, providing clues and evidence for future clinical applications of cell replacement therapy for liver disease.

## Additional file

Additional file 1: **Table S1.** Primers used in PCR (DOCX 33 kb)

## Abbreviations

AFP: α-Fetoprotein
ALB: Albumin
bFGF: Basic fibroblast growth factor
CCK8: Cell counting kit-8
CK19: Cytokeratin 19
Dlk1: Delta-like 1 homolog
EMT: Epithelial-to-mesenchymal transition
ESCs: Embryonic stem cells
G6P: Glucose-6-phosphatase
HGF: Hepatocyte growth factor
HSCs: Hepatic stem cells
HSDCs: Hepatic spheroid-derived cells
iPSCs: Induced pluripotent stem cells
OSM: Oncostatin M
PBS: Phosphate-buffered saline
TAT: Tyrosine amino transferase
TNF-α: Tumor necrosis factor-α
TO: Tryptophan-2,3-oxygenase
