# Supplementary material for: Distinct roles of Dlk1 isoforms in bi-potential differentiation of hepatic stem cells
Source: Stem Cell Res Ther. 2019 Jan 15;10:31. doi: 10.1186/s13287-019-1131-2 (PMC6334473; doi:10.1186/s13287-019-1131-2)
Supplement: Supplementary file 1 — Table S1. Primers used in PCR (DOCX 33 kb) [file 13287_2019_1131_MOESM1_ESM.docx]

**Table S1. Primers used in PCR.**

| **Gene** | **Primer** | **Sequence (5'-3')** |
| --- | --- | --- |
| *Gapdh* | Forward | CCGTGTTCCTACCCCCAATGT |
|  | Reverse | GATGCCTGCTTCACCACCTTC |
| *Alb* | Forward | TGGGTAACCTTTCTCCTCCTCC |
|  | Reverse | CACTCTTGTGTGCTTCTCGGC |
| *G6p* | Forward | CATCAATCTCCTCTGGGTGGC |
|  | Reverse | CGTTGCTGTAGTAGTCGGTGTCC |
| *To* | Forward | TTTACACAGCCGAGTACAGTGACAG |
|  | Reverse | TCCAGGATTGGACCAAAACATC |
| *Ck19* | Forward | ACCCTCCCGAGATTACAACCAC |
|  | Reverse | CAAGGCGTGTTCTGTCTCAAAC |
| *Pparγ2* | Forward | TCTTCCATCACGGAGAGGTC |
|  | Reverse | GATGCACTGCCTATGAGCAC |
| *Opn* | Forward | CTTTCACTCCAATCGTCCCTAC |
|  | Reverse | GCTCTCTTTGGAATGCTCAAGT |
| *Dlk1* | Forward | CGTGAGACCTTGACCGAGTC |
|  | Reverse | CTGGATGGATGGTGGATGA |
| *Cyclin D1* | Forward | GGGTGGGTTGGAAATGAAC |
|  | Reverse | TCCTCTCCAAAATGCCAGAG |
| *Cyclin E1* | Forward | TCCACGCATGCTGAATTATC |
|  | Reverse | TTGCAAGACCCAGATGAAGA |
| *Cyclin A2* | Forward | GTGGTGATTCAAAACTGCCA |
|  | Reverse | AGAGTGTGAAGATGCCCTGG |
| *Cyclin B1* | Forward | GGCTTGGAGAGGGATTATCA |
|  | Reverse | ACCAGAGGTGGAACTTGCTG |
| *Tat* | Forward | ATAGGTTTGGCTGCTTGGAGAC |
|  | Reverse | GAACATCCAGCACTGAAGGTAGG |
| *Notch1* | Forward | GAGGTCAACGAGTGCAACAGTAAC |
|  | Reverse | CTCCACCCAGGGGCACAGTCAC |
| *Notch2* | Forward | GACTGCCAATACTCCACCTCT |
|  | Reverse | CCATTTTCGCAGGGATGAGAT |
| *Jag1* | Forward | CCTCGGGTCAGTTTGAGCTG |
|  | Reverse | CCTTGAGGCACACTTTGAAGTA |
| *Jag2* | Forward | TTCTGTGACGAGTGTGTCCC |
|  | Reverse | GCGCAGAGGTATTGGTCAGG |
| *Hes1* | Forward | CCAGCCAGTGTCAACACGA |
|  | Reverse | AATGCCGGGAGCTATCTTTCT |
| *α-SMA* | Forward | GGACGTACAACTGGTATTGTGC |
|  | Reverse | TCGGCAGTAGTCACGAAGGA |
| *Vimentin* | Forward | CGGCTGCGAGAGAAATTGC |
|  | Reverse | CCACTTTCCGTTCAAGGTCAAG |
